# W*_x_*NbMoTa Refractory High-Entropy Alloys Fabricated by Laser Cladding Deposition

**DOI:** 10.3390/ma12030533

**Published:** 2019-02-11

**Authors:** Qingyu Li, Hang Zhang, Dichen Li, Zihao Chen, Sheng Huang, Zhongliang Lu, Haoqi Yan

**Affiliations:** 1 State Key Laboratory for Manufacturing Systems Engineering, Xi’an Jiaotong University, Xi’an 710049, China; liqingyu9206@126.com (Q.L.); dcli@mail.xjtu.edu.cn (D.L.); chenzihao18@stu.xjtu.edu.cn (Z.C.); ahhuangsheng@126.com (S.H.); zllu@mail.xjtu.edu.cn (Z.L.); yan21601122@163.com (H.Y.); 2 School of Mechanical Engineering, Xi’an Jiaotong University, Xi’an 710049, China

**Keywords:** W***_x_***NbMoTa, refractory high-entropy alloy, laser cladding deposition, rapid solidification

## Abstract

W***_x_***NbMoTa refractory high-entropy alloys with four different tungsten concentrations (***x*** = 0, 0.16, 0.33, 0.53) were fabricated by laser cladding deposition. The crystal structures of W***_x_***NbMoTa alloys are all a single-phase solid solution of the body-centered cubic (BCC) structure. The size of the grains and dendrites are 20 μm and 4 μm on average, due to the rapid solidification characteristics of the laser cladding deposition. These are much smaller sizes than refractory high-entropy alloys fabricated by vacuum arc melting. In terms of integrated mechanical properties, the increase of the tungsten concentration of W***_x_***NbMoTa has led to four results of the Vickers microhardness, i.e., *H_v_* = 459.2 ± 9.7, 476.0 ± 12.9, 485.3 ± 8.7, and 497.6 ± 5.6. As a result, NbMoTa alloy shows a yield strength (σ_b_) and compressive strain (ε_p_) of 530 Mpa and 8.5% at 1000 °C, leading to better results than traditional refractory alloys such as T-111, C103, and Nb-1Zr, which are commonly used in the aerospace industry.

## 1. Introduction

With the rapid development of materials and manufacturing technologies in the aerospace industry, the established macroscopic thermal protection theory and the existing traditional types of the refractory alloy are difficult to meet the harsh requirements of the advanced aerospace industry. Traditional alloy design is based on a metallic element with a mass fraction of more than 50% and the comprehensive properties of the alloys are improved by adding trace elements. This system of alloy design has reached the bottleneck after thousands of years of development.

In 2004, Taiwan scholar professor Yeh JW and his team [1] proposed the concepts of multi-principal element alloys (MPEAs) and high-entropy alloys (HEAs). Generally, HEAs can be defined as a simple and disordered structure of solid solution mixed by multi-elements of 5%–35% atom ratio, showing specific characteristics, such as the high-entropy effect in thermodynamics and hysteresis diffusion effect in dynamics [2,3]. These characteristics then contribute to the advantages of HEAs in such aspects as high-temperature resistance [4], high strength and ductility [5,6], corrosion and radiation resistance [7,8], providing more possible applications in the aerospace industry.

In the research field of refractory HEAs, researchers have conducted a series of studies on the different refractory HEAs in strength and ductility at small scales [9,10], the relationship between grain size and mechanical properties [11], thermodynamic properties [12] and the interplay among lattice distortions, vibrations, and phase stability [13]. In 2011, Senkov, O.N. [14] produced the refractory HEAs with equiatomic concentrations, WNbMoTa and WNbMoTaV, via vacuum arc melting (VAM) for the first time, obtaining a single-phase body-centered cubic (BCC) structure. Later on, he [4] found that WNbMoTa and WNbMoTaV HEAs have better high-temperature yield strength than traditional superalloys, namely Inconel 718 and Haynes 230. In 2017, after adding the equal mole titanium element, Han [15] improved the ductility of WNbMoTa and WNbMoTaV through VAM at room temperature. As a result, existing research is mainly aimed at revealing the nature or the characteristics of HEAs, instead of being driven by engineering applications.

At present, refractory HEAs are mainly produced by the methods of VAM or powder metallurgy (PM). Traditional manufacturing methods have difficulties in forming the HEAs with a large size, a complex structure, and a variable composition. The technology of laser cladding deposition (LCD) is the process that fuses the metal powders from points to layers and finally fabricates the parts according to the three-dimensional model data. As an emerging technology, it has many unparalleled advantages in forming refractory HEAs. One the one hand, refractory alloys can be melted rapidly due to the high energy density of the laser, thus not limiting the forming size and structure. On the other hand, the functionally gradient structure of the HEAs can be manufactured in any direction of the parts by controlling the feeding rate of the different hopper, which can realize the macrostructure and micro metallurgy synchronous manufacturing [16]. In 2016, Dobbelstein [17] prepared WNbMoTa HEA by means of remelting in the process of LCD, analyzing its composition uniformity and macroscopic segregation. Then, in 2018, Zhang [18] formed WNbMoTa HEA through selective laser melting (SLM) and carried out the simulation of the finite difference-finite element (FD-FE) coupling in the SLM forming process.

Special requirements of “high strength, low density” are put forward due to the severe environment in aerospace. The high density of the tungsten element limits the application of the refractory HEA with equiatomic concentrations, WNbMoTa, in the aerospace industry. Presented in current work with the tungsten mole of 0%, 5%, 10% and 15%, and the remaining elements of the equal mole, refractory alloy materials were formed by LCD, respectively. The purpose of the present work is to recognize the relationships between mechanical behavior and the content of tungsten in the WNbMoTa HEAs and to provide a feasible scheme for the possibility of large-size and complex shaped gradient structures in the aerospace application.

## 2. Experimental Procedures

**Figure 1:**
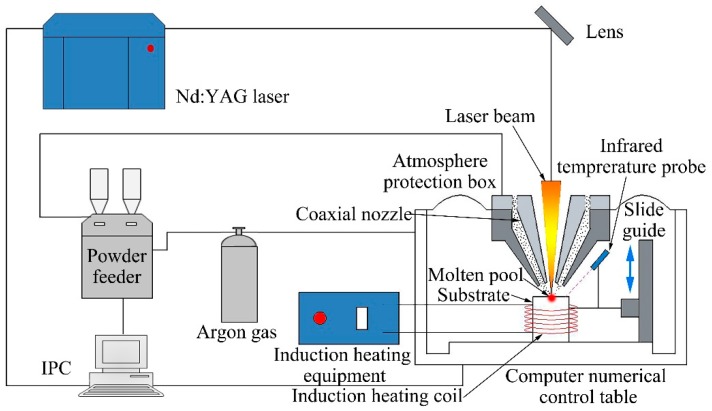
Schematic of the forming process of laser cladding deposition (LCD).

The four W***_x_***NbMoTa HEAs are referred to as ***x*** = 0, 0.16, 0.33 and 0.53, respectively. The deposition of the four different tungsten mole alloys were fabricated by using the LCD-1000-a type of the coaxial LCD system, which was independently designed and developed by the State Key Laboratory for Manufacturing Systems Engineering at Xi’an Jiaotong University. The system was equipped with two powder feeders, a 25 kW intermediate frequency induction heating auxiliary device and a standard, industrial pulse JK802/1002 Nd:YAG laser (JK Lasers, Rugby, UK) with 1000 W maximum power, which had a spot diameter of 500 μm at a central emission wavelength of 1064 nm. The diagram of the LCD forming process is shown in Figure 1.

**Figure 2:**
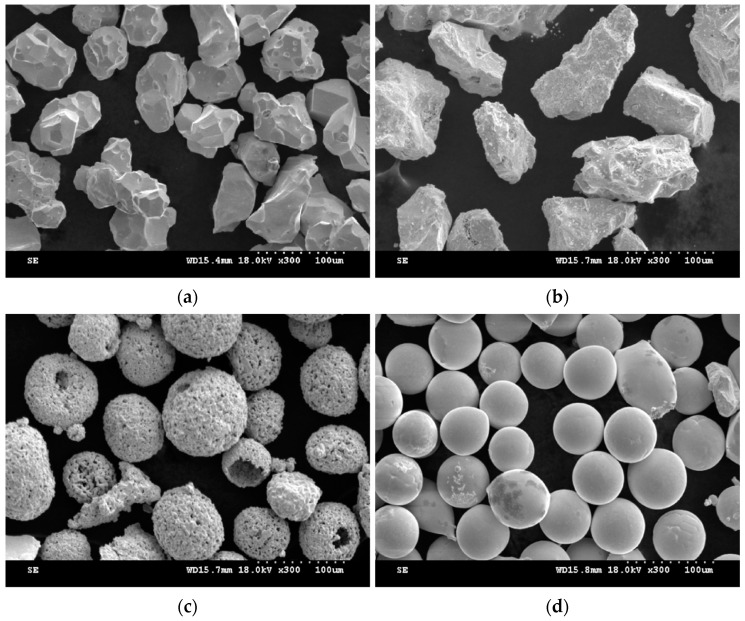
Scanning electron microscope (SEM) images of four metal powders. (**a**) Tungsten powders, (**b**) niobium powders, (**c**) molybdenum powders, (**d**) tantalum powders.

All experimental materials, including particles with sizes ranging from 45 μm to 125 μm elemental tungsten (99.5 wt.%), niobium (99.78 wt.%), molybdenum (99.84 wt.%), tantalum (99.54 wt.%) metal powders and Φ 20 mm × 80 mm of pure molybdenum substrates, were provided by Beijing AMC Powder Metallurgy Technology Co., Ltd. (Beijing, China). The scanning electron microscope photos of the four powders are shown in Figure 2 and the basic physical properties of the four elements are shown in Table 1.

The elemental metal powders of the four different tungsten mole alloys were accurately measured by the electronic, analytical balance before the experiment. Powders were mixed for 4 h via SYH three-dimensional motion mixer series to ensure that they were mixed evenly. Then, the mixed powders were put into a vacuum oven at 120 °C to dry for 8 h in order to remove moisture and enhance the liquidity of the powder. The atmosphere chamber was filled with Argon gas to ensure that the oxygen content of the environment was lower than 80 ppm for the purpose of protecting the powders from being oxidized during the forming process. The LCD processing parameters in the experiments after optimizing were 565 W laser power, 8 mm/s scanning speed, 0.08 mm thickness of the deposited layer, and 600 °C induction heating temperature. The substrates and the formed area were heated during the forming process to reduce the internal stress.

The crystal structures of the four different tungsten mole alloys were identified on the cross-section surfaces, using Bruker D8 Advanced A25 X-ray diffraction (XRD, Bruker AXS GmbH, Karlsruhe, Germany) equipment. Taking advantage of a METTLER TOLEDO XS105 (Mettler-Toledo GmbH, Zurich, Switzerland) analytical balance through the drainage method, the densities of the alloys were measured. Microstructures were analyzed with the use of a TESCAN MIRA3 LMH scanning electron microscope (SEM, TESCAN, a.s., Brno-Kohoutovice, Czech Republic) equipped with a backscatter electron (BSE) and an energy dispersive spectroscopy (EDS) detector. The sizes and orientations of the grains were determined by the electron back-scatter diffraction (EBSD) using an SU3500 SEM (Techcomp (China) Ltd, Beijing, China). Vickers microhardness was measured on polished longitudinal-section surfaces using an HXD-2000TMSC/LCD tester (Shanghai Taiming Optical Instrument CO., Ltd, Shanghai, China) and the test parameter was applied for 30 s under a 500 g load. Before the compression mechanical performance testing, the samples were cut into pieces that were 4 mm in diameter and 6 mm in height, giving an aspect ratio of 1.5. The room temperature compression test was conducted at 25 °C by a Sans CMT4304 multi-function static experiment machine (MTS Systems (China) CO., Ltd, Shenzhen, China) at a strain rate of 0.001 s^−1^, and the high temperature compression test was conducted at 1000 °C by Gleeble 3500 equipment. In high temperature compression, the chamber was evacuated to 10^−3^ torr. The sample was heated to 1000 °C in 3.5 min, soaked at this temperature for 15 min, and then compressed at a strain rate of 0.001 s^−1^.

## 3. Results and Discussions

**Figure 3:**
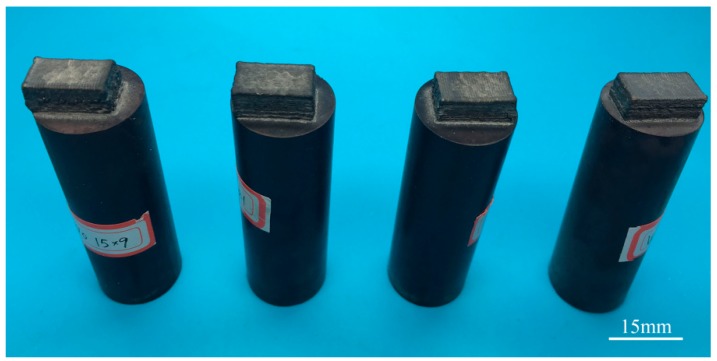
Macroscopic photograph of the W***_x_***NbMoTa alloys.

The W***_x_***NbMoTa HEAs were fabricated by LCD, shown in Figure 3. NbMoTa, W***_0.16_***NbMoTa, W***_0.33_***NbMoTa and W***_0.53_***NbMoTa ranged from left to right. Four groups of samples deposited by LCD had a rectangular geometry with the size of 15 mm × 9 mm × 6 mm.

### 3.1. Chemical and Phase Composition

The composition of the alloys, determined by EDS detector, is reported in Table 2. It can be found that the composition of each alloy is close to the ratio of design, and the biggest deviation is the mole of niobium in alloy 3, up to 2.6%. It shows that the composition uniformity is ensured and there is no obvious macroscopic burning loss to the elements during the process of LCD.

**Figure 4:**
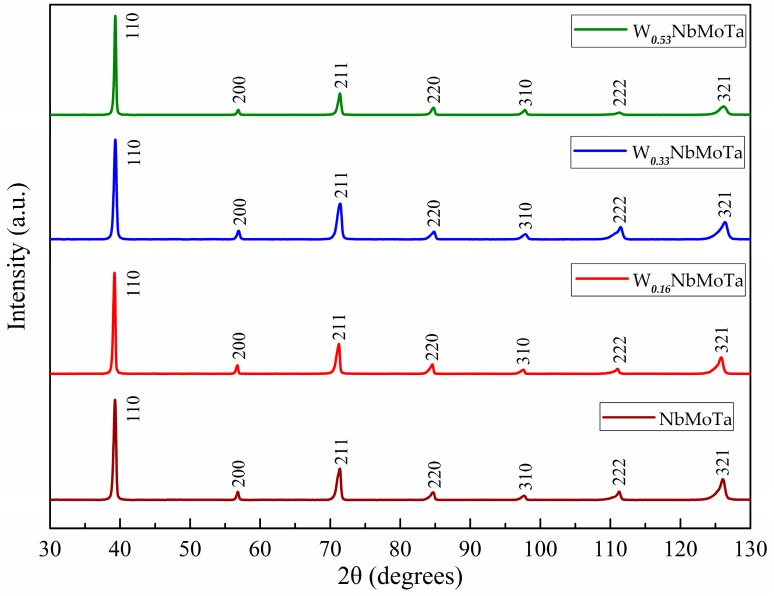
X-ray diffraction (XRD) patterns of the W***_x_***NbMoTa alloys.

The phase compositions of W***_x_***NbMoTa HEAs were checked by XRD patterns as shown in Figure 4. All peaks on these X-ray patterns have been indexed and are congruent with a single BCC phase. Meanwhile, there is no alternative phase found under the analysis of the XRD result. This result indicates that these elements could still form the same BCC structures in spite of the varied lattice parameters of the four elements. Four different tungsten mole alloys fabricated by LCD all have a single-phase BCC crystal structure. Due to the texture effects caused by the grains within the X-ray excited volume, the same peaks in the four alloys have shown the different relative intensities.

Yeh [1] proposed that HEAs should contain at least five elements, and that the entropy of mixing (*ΔS_mix_*) is the main factor that promotes the formation of a multicomponent solid solution. With further research, WNbMoTa [14], NbTiVZr [19] quaternary equiatomic alloys and the ZrNbHf [20] ternary equiatomic alloy are proven to be able to form typical HEAs. Therefore, the formation of the solid solution in HEAs systems can be predicted in terms of the atomic size difference (*δ*), enthalpy of mixing (*ΔH**_mix_***) and other factors in addition to the entropy of mixing (*ΔS_mix_*).

Atomic size difference (*δ*) is determined by:

(1) $$\delta =100\sqrt{\sum_{i=1}^{n}c_{i}{\left(1-\frac{r_{i}}{\sum_{j=1}^{n}c_{j}r_{j}}\right)}^{2}}$$

where *c_i_* and *c_j_* are the atomic fractions of the *i-*th and *j-*th components, *r_i_* and *r_j_* are the atom radii of the *i-*th and *j-*th components.

Entropy and enthalpy of mixing could be obtained by:

(2) $$\Delta S_{mix}=-R\overset{n}{\underset{1}{\sum }}c_{i}\ln c_{i}$$

(3) $$\Delta H_{mix}=\overset{n}{\underset{i=1,i\neq j}{\sum }}\Omega_{ij}c_{i}c_{j},\Omega_{ij}=4\Delta H_{AB}^{mix}$$

where *ΔS_mix_* is mixing entropy change, *ΔH_mix_* is mixing enthalpy mixing, *R* is constant gases, $\Delta H_{AB}^{mix}$ is the enthalpy change of the binary liquid alloy composed of the components A and B in the regular solution, which is calculated based on Miedema macroscopic model [21,22].

Zhang [3] proposed a new parameter to determine the ability of the solid solution formation of the multi-component alloys. It consists of *ΔS_mix_*, *ΔH_mix_* and can be defined as follows:

(4) $$\Omega =\frac{T_{m}\Delta S_{mix}}{\left|\Delta H_{mix}\right|}$$

(5) $$T_{m}=\sum_{i=1}^{n}c_{i}(T_{m}{)}_{i}$$

where *T_m_* is the average melting point of the alloy, *(T_m_)_i_* is the melting point of *i-th* component. Combined with *δ* and Ω parameters, statistical analysis has been made for a large number of known solid solution HEAs. Then, the criteria for forming staple HEAs solid solution are [23,24]:

***δ***% ≤ 6.6%, Ω ≥ 1.1 (6)

The relevant parameters of four different tungsten mole HEAs are shown in Table 3. The calculated parameters meet the criteria of solid solution formation and are consistent with the experimental XRD patterns.

### 3.2. Density and Microstructure

The density of the alloys measured by the drainage method is reported in Table 4. According to the theoretical density of the alloys, it can be calculated by the formula:

(7) $$\rho_{other}=\frac{\sum c_{i}A_{i}}{\sum \frac{c_{i}A_{i}}{\rho_{i}}}$$

where ***c_i_***, *A_i_* and *ρ*_i_ are the atomic fraction, atomic weight and density of element *i*, respectively, and calculated *ρ_theor_* value for the four different tungsten mole high-entropy alloys is also reported in Table 4.

**Figure 5:**
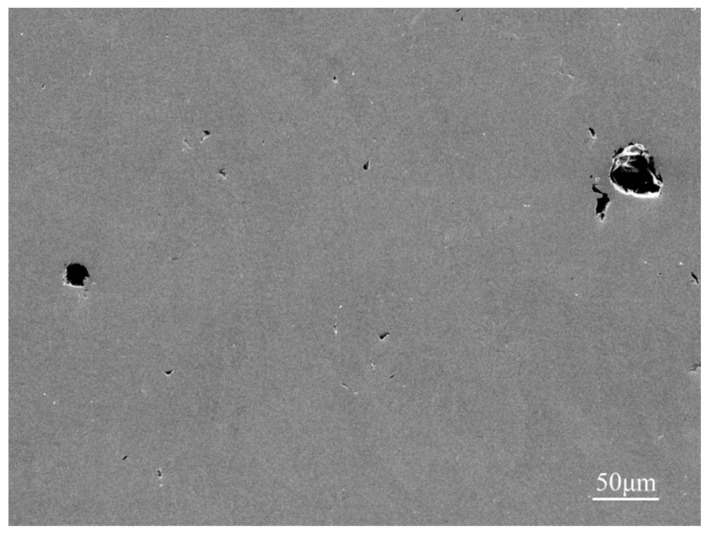
SEM image of the gas porosities with spherical shape in HEAs.

It can be seen that the experimental densities are lower than the theoretical densities in that many spherical gas porosities appear inside the alloys during the process of LCD, which is shown in Figure 5. In addition, the quality of the powders is different, owing to the different preparation methods of metal powders. Figure 1 shows that the mechanically pulverized tantalum powders were spheroidized through the technology of radio frequency plasma spherification, showing a good property in sphericity and granule density, while the tungsten and niobium powders were not spheroidized after mechanical grinding, leading to a higher granule density but a poor sphericity. Molybdenum powders were prepared through granulation reunion. The sphericity of molybdenum powders is much better than tungsten and niobium powders, but all of them are hollow, having nodular surfaces and containing significant amounts of pores.

**Figure 6:**
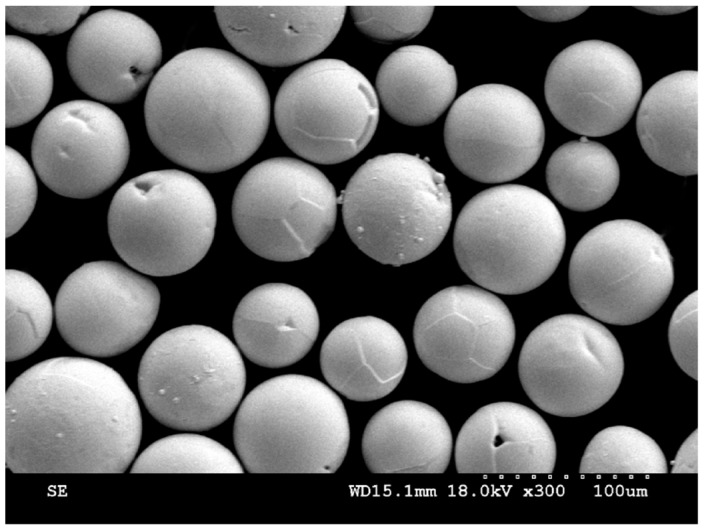
SEM image of the molybdenum powders with high granule density.

**Figure 7:**
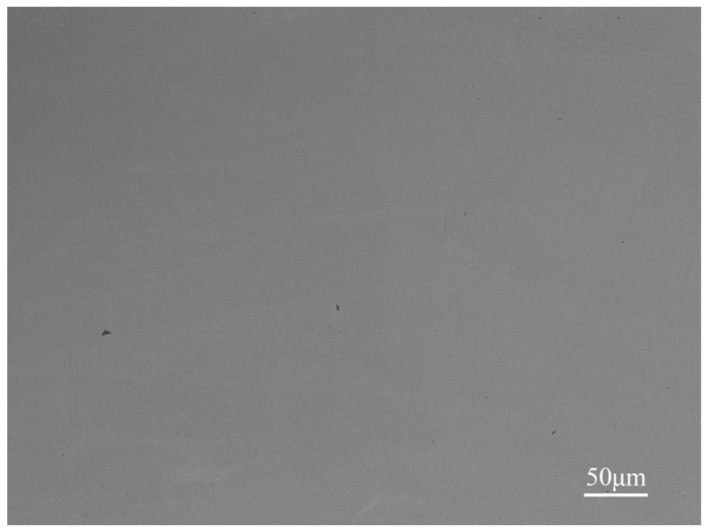
SEM image of the HEAs used powders with high granule density.

The studies have shown that the granule density of powders is the main factor affecting the gas porosities. Conclusions have been verified in Ti-6Al-4V, Inconel 718 and other powders’ forming process [25,26,27]. During the forming process, the protective Argon gas from the powder feed system is adsorbed in the pores of molybdenum powders, drawn into the forming part, forming a certain amount of spherical and randomly distributed gas porosities. The appearance of porosities has no correlation with microstructure features. In order to verify the above conclusions, the molybdenum powders with high granule density and good sphericity were used to replace that used in the previous experiment, shown in Figure 6. Then, the HEA was formed with the same process parameters to control a single variable. The SEM image of the formed sample is shown in Figure 7 and the porosities disappeared. This evidence strongly suggests that the granule density, instead of sphericity of powders, has a great influence on the defects of porosities. Particle pores may directly contribute to deposit porosities.

**Figure 8:**
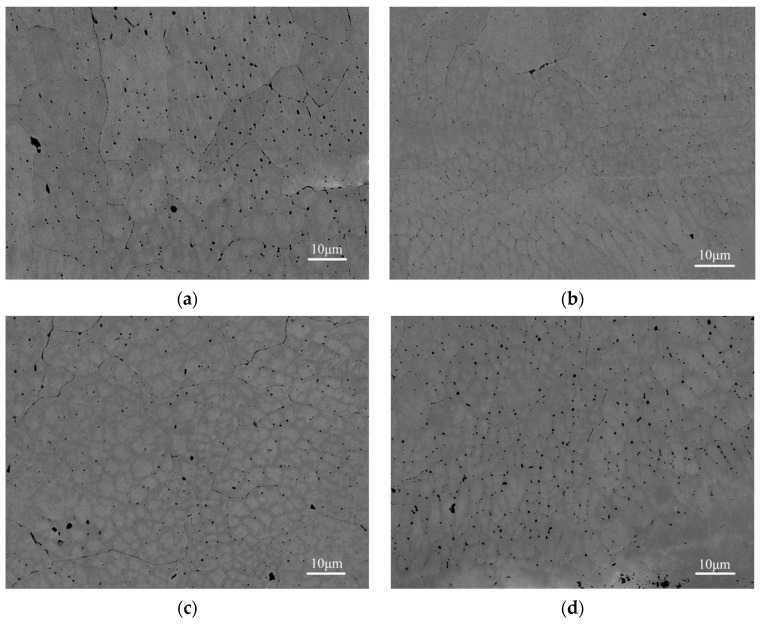
Backscatter electron (BSE) of polished longitudinal-sections of the W***_x_***NbMoTa HEAs. (**a**) NbMoTa, (**b**) W***_0.16_***NbMoTa, (**c**) W***_0.33_***NbMoTa, (**d**) W***_0.53_***NbMoTa.

**Figure 9:**
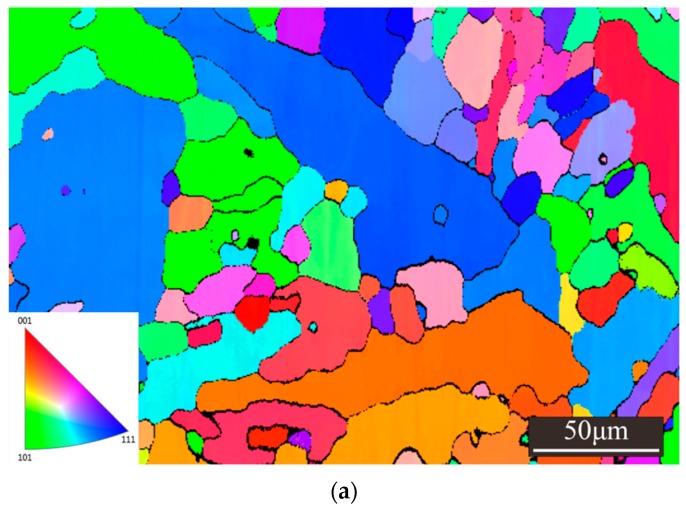

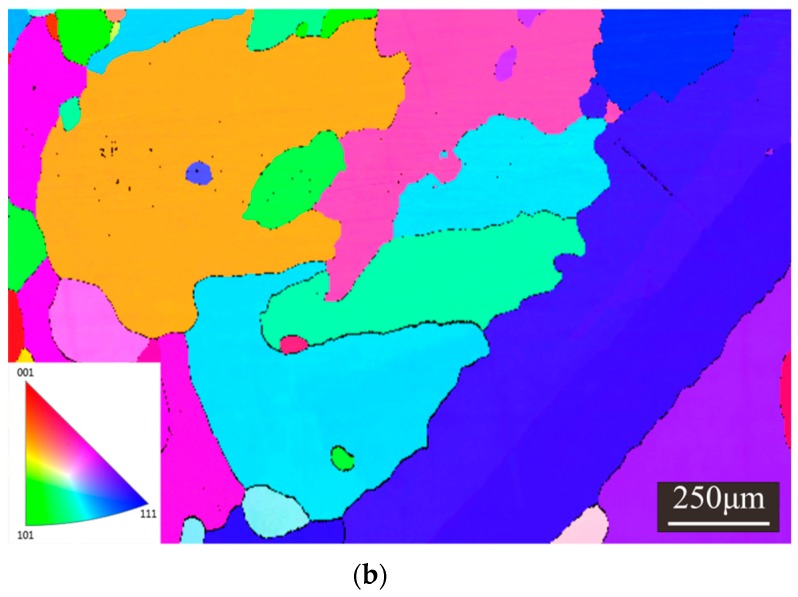
EBSD of polished longitudinal-sections of the NbMoTa HEAs. (**a**) Laser cladding deposition (LCD), (**b**) vacuum arc melting (VAM).

BSE images of the W***_x_***NbMoTa HEAs are shown in Figure 8. The microstructures of the W***_x_***NbMoTa are similar. The interior of the grain contains spheroidal dendrites (as the sub-structure of grain). Grains of about 20 μm in diameter and dendrites of about 4 μm in diameter can be seen. EBSD images of the NbMoTa HEAs formed in the process of LCD and VAM are shown in Figure 9. The grain size of NbMoTa fabricated by LCD is much smaller than that of NbMoTa fabricated by VAM and the growth way of grains is equiaxed crystals. Due to the characteristics of rapid solidification, the laser with high energy density fuses the metallic powders with a high melting point in a moment (10^−3^–10^−2^ s). The phase transition of powder materials from the liquid phase to the solid phase is so fast that it is easy to form fine grain, low constitutional segregation microstructure.

This is compared to the HEAs produced by vacuum arc melting. According to the Hall-Petch relation, the yield strength or microhardness of materials has a negative linear correlation with grain size, when the grain size of testing materials is not ultrafine (≥1 μm) [28,29,30,31,32]. In engineering manufacture, large yield strength or hardness of the material may be attained by decreasing the grain size, while the characteristics of rapid solidification in LCD facilitate the formation of fine grain. Compared with traditional VAM manufacturing, the yield strength and microhardness of alloys by LCD are usually much better.

### 3.3. Mechanical Properties

**Figure 10:**
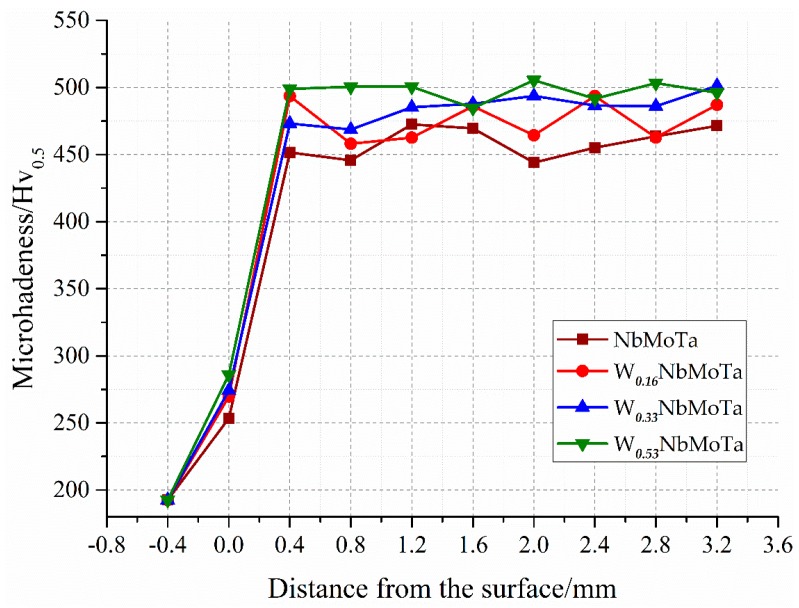
Microhardness of the W***_x_***NbMoTa HEAs fabricated in LCD.

The Vickers microhardness of the different tungsten mole HEAs were measured from the substrate to the metallurgical bonding surface, and then the cladding layer. With the increase of the cladding height, no obviously regular change was found in microhardness. The results are demonstrated below in Figure 10. The average values of microhardness are shown in Table 5. With the increase of the content of tungsten, the hardness shows an increasing tendency.

**Figure 11:**
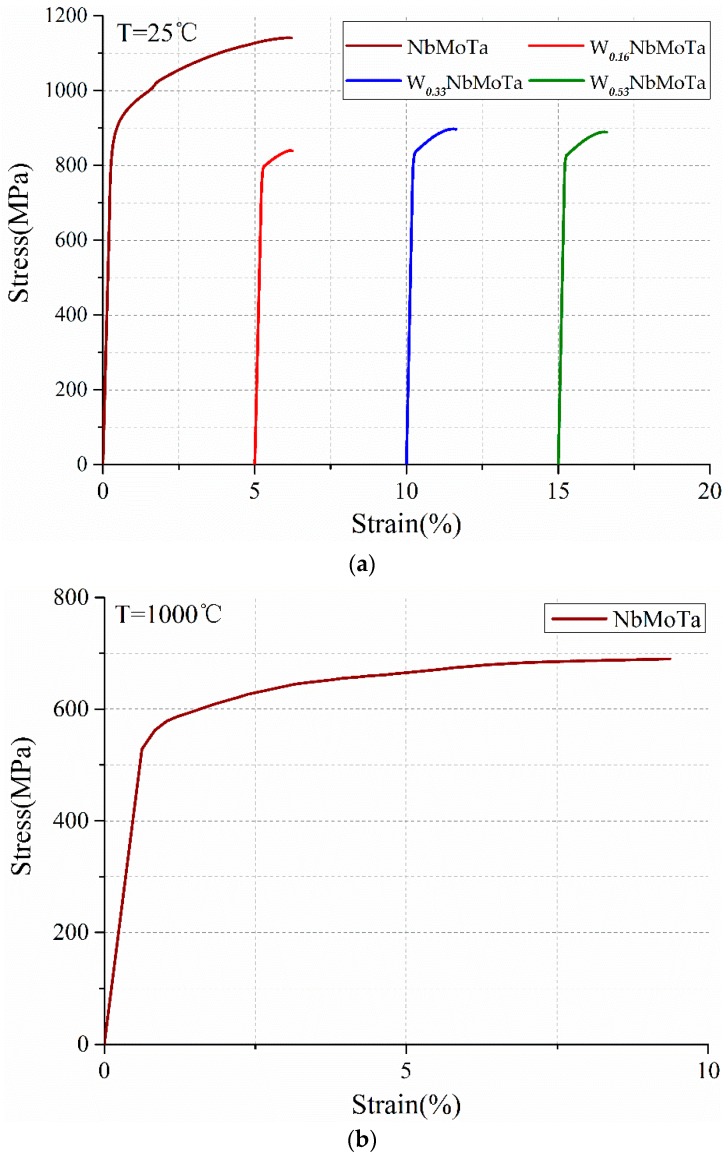
Compressive stress-strain curves of different temperature for the W***_x_***NbMoTa HEAs. (**a**) Room temperature, (**b**) high temperatures.

The bulk modulus of the W***_x_***NbMoTa (*x* = 0, 0.16, 0.33, 0.53) were measured to be 174 GPa, 182 GPa, 186 GPa and 192 GPa. The compressive stress-strain curve of the alloys at room temperature (T = 25 °C) is illustrated in Figure 11a. NbMoTa showed a yield strength (σ_b_), compressive strength (σ_m_) and compressive strain (ε_p_) of 874 MPa, 1140 MPa and 5.8%, respectively. The compressive strain was improved compared with the WNbMoTa HEAs by VAM (1.5%) [4], indicating that NbMoTa has certain machinability at room temperature. W***_0.16_***NbMoTa, W***_0.33_***NbMoTa and W***_0.53_***NbMoTa were characterized as brittle materials, and their maximum compressive strength (σ_b_) showed 840 MPa, 895 MPa, and 890 MPa.

The compressive stress-strain curve for the NbMoTa at 1000 °C is illustrated in Figure 11b, showing a yield strength (σ_b_), compressive strength (σ_m_) and compressive strain (ε_p_) of 530 MPa, 684 MPa and 8.5%, respectively. It reflects a better effect than such traditional refractory alloys, as T-111, Nb-1Zr and C103 are commonly used in aerospace industry [33], as shown in Table 6. The result indicates that NbMoTa fabricated by LCD shows potential for application in the aerospace industry.

## 4. Conclusions

The purpose of the work is to study the dependence of the yield strength of the refractory HEAs on their composition fabricated by LCD for the potential applications in aerospace industry. The following conclusions are drawn:

(1) The crystal structures of each W***_x_***NbMoTa (*x* = 0, 0.16, 0.33, 0.53) alloys are all a single-phase solid solution of the BCC structure analyzed by XRD.
(2) Due to the characteristic of rapid solidification, the size of the grains and dendrites on the microcosmic of W***_x_***NbMoTa refractory HEAs was 20 μm and 4 μm on average, smaller than that of the HEAs fabricated by VAM.
(3) The increase of the tungsten concentration of W***_x_***NbMoTa led to four results of the Vickers microhardness, i.e., *H_v_* = 459.2 ± 9.7, 476.0 ± 12.9, 485.3 ± 8.7, 497.6 ± 5.6, respectively.
(4) The NbMoTa alloy has a compressive strain (ε_p_) of 5.8% at room temperature and its yield strength (σ_b_), compressive strength (σ_m_) and compressive strain (ε_p_) of 530 MPa, 684 Mpa and 8.5% respectively at 1000 °C. The effects show better performance than many traditional refractory metals such as T-111, Nb-1Zr, and C103, which are commonly used in aerospace.
(5) The content of tungsten has no effect on the formation of a single-phase solid solution and the microstructure of the HEAs. In terms of mechanical behavior, the microhardness shows an increasing tendency with the increase of the content of tungsten. As a result, the yield strength and plasticity of the W-free alloy is improved compared with alloys containing tungsten at room temperature.

In this study, with excellent yield strength at high temperature, the NbMoTa alloy shows potential for application in aerospace industry. The present work provides a theoretical basis for LCD manufacturing of aerospace parts of refractory HEAs with a large size, complex structure, and variable composition.

## Figures and Tables

**Table 1:** The basic physical parameters of the four materials used in the alloy.

Metallic Element.	W	Nb	Mo	Ta
Relative Atomic Mass, u	183.84	92.9	95.94	180.9
r, Å	1.37	1.43	1.36	1.43
*ρ*, g/cm^3^	19.35	8.57	10.2	16.65
*Hv*	350	135	156	89
*T_m_*, K	3695	2750	2896	3290

**Table 2:** Chemical composition (in at.%) of four refractory alloys produced by LCD.

Alloy ID/element	W	Nb	Mo	Ta
NbMoTa	0%	31.26%	32.90%	35.84%
W*_0.16_*NbMoTa	5.80%	29.76%	30.86%	33.58%
W*_0.33_*NbMoTa	12.42%	27.40%	30.01%	30.17%
W*_0.53_*NbMoTa	14.95%	26.80%	27.62%	30.63%

**Table 3:** The parameters, *δ*, *ΔS_mix_*, *ΔH_mix_*, *T_m_* and Ω for W***_x_***NbMoTa high-entropy alloys (HEAs).

Parameter/Alloy ID	NbMoTa	W*_0.16_*NbMoTa	W*_0.33_*NbMoTa	W*_0.53_*NbMoTa
*δ*, %	2.334	2.359	2.365	2.364
*ΔS_mix_*, J·mol^−1^·K^−1^	9.14	10.33	10.93	11.28
*ΔH_mix_*, J·mol^−1^	−4.67	−5.22	−5.69	−6.07
*T_m_*, K	2979	3015	3050	3086
Ω,	5.83	5.96	5.86	5.74

**Table 4:** Theoretical and experimental densities of the W***_x_***NbMoTa HEAs.

Alloy ID/Density	Theoretical Density; g/cm^3^	Experimental Density; g/cm^3^
NbMoTa	11.913	10.486
W*_0.16_*NbMoTa	12.205	10.572
W*_0.33_*NbMoTa	12.595	10.634
W*_0.53_*NbMoTa	12.940	11.044

**Table 5:** Experimental *H_v_* and theoretical *H_v_* values of the W***_x_***NbMoTa HEAs.

*Hv*/Alloy ID	NbMoTa	W*_0.16_*NbMoTa	W*_0.33_*NbMoTa	W*_0.53_*NbMoTa
Experimental *H_v_*	459.2 ± 9.7	476.0 ± 12.9	485.3 ± 8.7	497.6 ± 5.6

**Table 6:** The comparison of high temperature (1000 °C) performance of aerospace materials.

Alloy ID	Yield Strength at 1000 °C/MPa
Nb-1Zr	113
C103	144
ODS-MA754	212
Mo-14Re	371
T111	505
NbMoTa	530
